# Differentially-dimensioned furrow formation by zygotic gene expression and the MBT

**DOI:** 10.1371/journal.pgen.1007174

**Published:** 2018-01-16

**Authors:** Yi Xie, J. Todd Blankenship

**Affiliations:** Department of Biological Sciences, University of Denver, Denver, CO, United States of America; New York University, UNITED STATES

## Abstract

Despite extensive work on the mechanisms that generate plasma membrane furrows, understanding how cells are able to dynamically regulate furrow dimensions is an unresolved question. Here, we present an in-depth characterization of furrow behaviors and their regulation in vivo during early *Drosophila* morphogenesis. We show that the deepening in furrow dimensions with successive nuclear cycles is largely due to the introduction of a new, rapid ingression phase (Ingression II). Blocking the midblastula transition (MBT) by suppressing zygotic transcription through pharmacological or genetic means causes the absence of Ingression II, and consequently reduces furrow dimensions. The analysis of compound chromosomes that produce chromosomal aneuploidies suggests that multiple loci on the X, II, and III chromosomes contribute to the production of differentially-dimensioned furrows, and we track the X-chromosomal contribution to furrow lengthening to the *nullo* gene product. We further show that checkpoint proteins are required for furrow lengthening; however, mitotic phases of the cell cycle are not strictly deterministic for furrow dimensions, as a decoupling of mitotic phases with periods of active ingression occurs as syncytial furrow cycles progress. Finally, we examined the turnover of maternal gene products and find that this is a minor contributor to the developmental regulation of furrow morphologies. Our results suggest that cellularization dynamics during cycle 14 are a continuation of dynamics established during the syncytial cycles and provide a more nuanced view of developmental- and MBT-driven morphogenesis.

## Author summary

One of the primary events that must occur repeatedly throughout a complex animal’s lifetime is the ingression of a plasma membrane furrow. Furrow formation and ingression are requisite elements of cell division, and drive the physical separation of one cell into two cells. However, the mechanisms that permit an embryo to change the length and size of a furrow are unclear. Here, we show that a combination of higher ingression rates and longer duration phases drive changes in furrow dimensions through the introduction of a new ingression phase. These changes are driven by the embryo’s own genome, and suggest that zygotic transcription controls organismal form at an earlier time point than previously appreciated. Additionally, the failure to properly lengthen furrows as development proceeds causes defects in chromosome segregation during cell division and results in massive genomic instability. Our data demonstrate the importance of the dynamic regulation of furrow dimensions to organismal form and viability.

## Introduction

Furrow ingression is an obligatory step in animal cells during cell division, and is a critical mechanism that underlies the ability of animal cells to divide and provide new cells for tissue homeostasis and development. While much of our knowledge of how plasma membrane furrows form and ingress comes from studies in isolated tissue culture cells, cells in different tissues and developmental contexts complete cell division at different rates, and possess different constraints in the resources available to them [reviewed in 1, 2]. Additionally, cells vary greatly in size and shape, suggesting that regulation of furrow dynamics in response to mechanical or regulatory cues occurs.

*Drosophila* embryogenesis initiates with a single nucleus that begins 13 cycles of rapid nuclear replication and division in an acellular syncytium. The first nine rounds of nuclear divisions occur deep in the yolk, with nuclei migrating to the periphery of the embryo at cycle 10. At this point, the density of nuclei and their arrangement in a common cortical plane requires four cycles of transient plasma membrane furrow formation (syncytial division cycles 10–13) to adequately partition mitotic figures and ensure genomic stability [3–5]. At cycle 14 (cellularization), plasma membrane furrows permanently encapsulate individual nuclei, resulting in a monolayered epithelium [6–8]. It is in these early furrow-forming processes that rapid, morphogenetic changes occur in furrow structure and dimensions. As cycles 10–14 proceed, the furrows are sequentially narrower and more regular, and furrows extend deeper basally, generating greater nuclear separation.

Intriguingly, it is in this same period of rapid furrow morphogenesis that the zygotic genome becomes transcriptionally active, and a hand-off in genetic regulation occurs from maternal to zygotic control (also called the MBT, or midblastula transition). While the MBT is often classically-defined by events that occur during cycle 14, zygotic gene expression can be detected as early as cycle 2 at a few isolated loci [9], and several hundred genes become actively transcribed during cycles 8–13 [10–13]. Two large sets of early expressed genes have been identified that are dependent on either Zelda or GAGA-factor transcription factors [14–18]. While GAGA-factor dependent transcripts are expressed relatively late in the MBT, Zelda appears to act earlier and is often associated with expression prior to cycle 14. In addition to zygotic activation, maternal gene products are gradually removed [19–21]. This coordination of zygotic gene activation and maternal gene decay ensures the proper, wild-type development of the *Drosophila* embryo. However, the function of these early transcripts as well as the clearance of maternal products in directing morphogenesis prior to cycle 14 has not been clear. Here, we examine the extent to which the ingression of differentially dimensioned furrows in the early embryo is a result of MBT-based developmental regulation of furrow dynamics.

To perform the first, comprehensive time-resolved measurements of wild-type syncytial furrow dynamics in the early *Drosophila* embryo, we used live 4D imaging to follow furrow formation and retraction behaviors. We find that a new ingression phase (Ingression II) follows the stabilization phase of cycles 12 and 13 and helps to drive the four-fold lengthening of furrows that occurs between cycles 10 to 13. While Ingression I is largely dependent on maternal gene products, Ingression II requires zygotic gene transcription. Either genetic or pharmacological blockage of new transcript production results in a complete loss of Ingression II and a loss of furrow lengthening. Through the use of compound chromosomal fly lines, we show that multiple loci on the X, II, and III chromosomes are required for the developmental production of differentially dimensioned furrows. We also find that cell cycle regulation of interphase and mitotic periods, which occurs during cycles 10–14, is permissive for furrow regulation, but is not strictly deterministic. Thus, we propose that direct developmental regulation by zygotic products is responsible for furrow lengthening and the changes in furrow dimensions in the early *Drosophila* embryo.

## Results

### Formation of differentially-dimensioned furrows during early syncytial development

**Fig 1:**
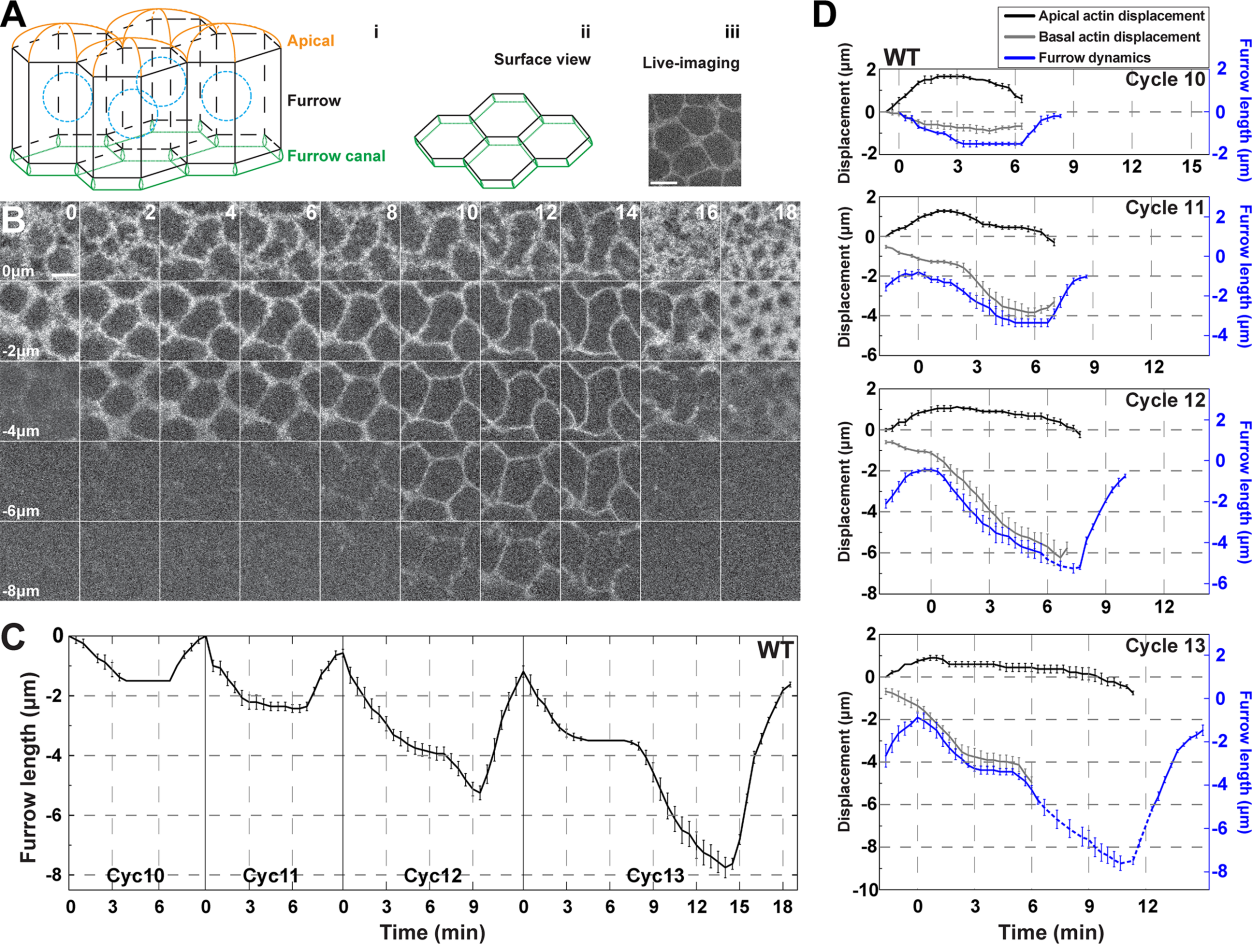
Developmental regulation of furrow dimensions and morphologies in the early *Drosophila* embryo. (A) Model of syncytial furrows indicating the apical region, furrow, and furrow canal (i). A planar view of the furrow canal regions is also shown (ii), as well as live-imaging data (iii). Scale bar = 5 μm (B) Still images of furrow dynamics from live-imaged cycle 13 embryos (Gap43:mCh) at t = 0min, 2min, 4min, 6min, 8min, 10min, 12min, 14min, 16min, and 18min, and z-planes at 0, -2, -4, -6, and -8μm. z = 0μm is most apical plane, z = -8μm is most basal. Scale bar = 5 μm (C) Wild type furrow dynamics from cycle 10–13 (cycle 10: n = 4; cycle 11: n = 7; cycle 12 and 13: n = 8). (D) Wild type apical actin displacement (GFP:moeABD, black curve), basal actin displacement (grey curve), and furrow dynamics (Gap43:mCh, blue curve) from cycle 10–13 (n = 4). Dashed blue curves are supplemented from independent data for the out-of-view furrow dynamics. Basal actin displacement curves (grey) end due to actin disbandment at anaphase (cycle 10–12) or to actin moving beyond the field of view (cycle 13).

At nuclear cycle 10 the majority of nuclei have migrated to adopt a subapical position beneath the cortex. At this point, five cycles of plasma membrane furrow formation begin, with furrows of characteristic differing lengths forming in each cycle. To examine this more closely, we imaged living embryos expressing markers of the plasma membrane (Gap43:mCherry) and chromosomes (Histone:GFP), which permits the imaging and quantitation of individual furrow dynamics, as well as the tracking of cell cycle behaviors. The measured, true furrow region is defined as the furrow length that stretches from where apical caps meet to the end of the furrow canal (Fig 1A and 1B). To establish furrow dynamics without the introduction of averaging artifacts due to slightly variable cycle durations between individual embryos, we aligned furrow formation and retraction measurements from each embryo and nuclear cycle with the onset of anaphase, as indicated by the Histone:GFP marker.

Similar to previous results [5, 22], furrow formation in wild-type embryos is highly reproducible, with furrows growing in length with each succeeding nuclear cycle. However, with greater temporal resolution, individual cycle dynamics became clearer (Fig 1B and 1C). At cycle 10, short furrows of only 1.5μm form in ~4 minutes. Furrows then deepen progressively with each cycle by ~2μm until cycle 14 (Fig 1C). At cellularization (interphase of cycle 14), deep furrows of ~30μm package nuclei into individual plasma membrane compartments (S1A Fig). Furrow morphologies also become sharper and more regular with each cycle (Fig 1B; S1B Fig).

### Introduction of a new ingression phase to direct furrow lengthening

**Fig 2:**
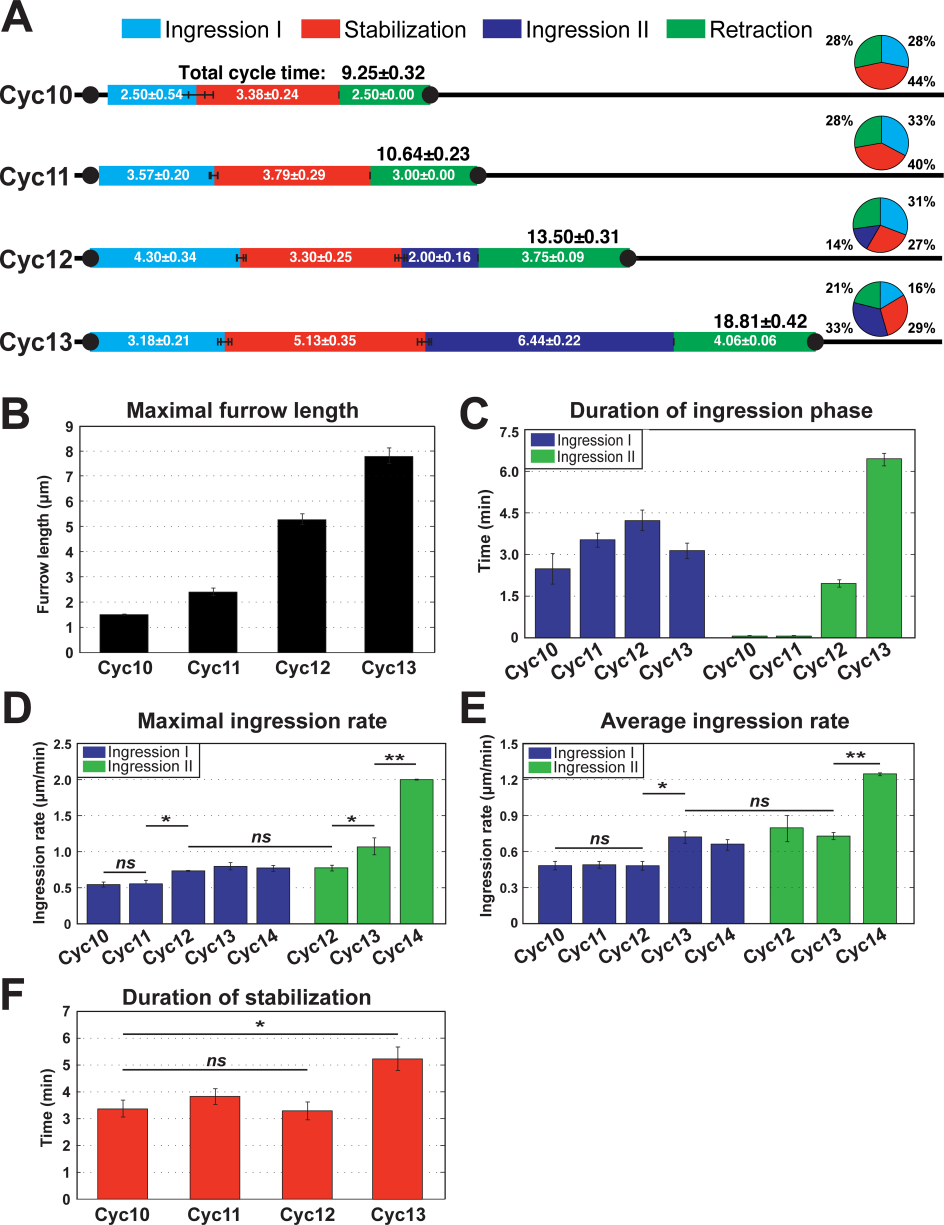
Quantitation of furrow dynamics and ingression rates in WT embryos. (A) Phases of furrow dynamics from cycle 10–13 in WT. The duration of each phase as well as total cycle times are indicated. The pie chart shows the percentage of duration of each phase for the identified cycle. (B) Maximal furrow length in WT embryos for cycles 10–13 (n≥4). (C) Duration of ingression phase in WT cycles (n≥4). (D) Maximal WT furrow ingression rate (n≥4). *:p<0.05; **: p<0.005; *ns*: not significant. Maximal rates are calculated from a 2 minute rolling window. (E) Average WT furrow ingression rate during Ingression I or Ingression II (n≥4). *:p<0.05; **: p<0.005; *ns*: not significant. (F) Duration of stabilization phase in cycle 10–13 (n≥4). *:p<0.05; *ns*: not significant.

The lengthening of furrows raises the question: what are the important formative events that drive changes in furrow dimensions? Between cycles 10 and 11, furrow invagination is driven by a single, initial ingression phase (Ingression I), which is then followed by a period of stable furrow lengths and eventual furrow retraction (Fig 1C and Fig 2A). Furrow morphologies are also less defined, with broader, more amorphous furrow tips during Ingression I of all cycles (0–4 min of Fig 1B). The deepening in furrow dimensions between cycle 10 and 11 is driven by a slightly enhanced time of the Ingression I phase, from 2.5 min to 3.6 min (Fig 2A–2C).

Interestingly, as furrows begin to reach depths 3x (cycle 12) and 5x (cycle 13) greater than initial furrow lengths at cycle 10, a different dynamic is initiated. Furrow lengthening during cycles 12 and 13 becomes dependent on the introduction of a new ingression phase (Ingression II) (Fig 1B and 1C; Fig 2A and 2B). Ingression II is not apparent in cycles 10 and 11, but then drives furrow invagination for 2.0 min during cycle 12 and for 6.2 min during cycle 13 (Fig 2A and 2C). By cycle 13, Ingression II encompasses 33% of the total cycle time and contributes 4.3μm of greater furrow length (Fig 2A–2C). Intriguingly, when Ingression II is introduced at cycle 12, it lacks robustness, with only 58% of embryos (n = 12 embryos) showing a discrete Ingression II (S1C Fig). However, by cycle 13, Ingression II is robust with all embryos displaying a second ingression that more than doubles furrow lengths as compared to Ingression I.

Notably, the maximum and average ingression rates of Ingression I from cycle 10–14 are largely similar, although the average rate increases by an additional 0.6x at cycle 13 (Fig 2D and 2E). However, the maximum ingression rate of Ingression II increases exponentially during cycles 12–14, thus driving greater furrow depths (Fig 2B). Ingression II during cellularization corresponds to fast phase, and has a 2.4-fold faster maximum rate than cycle 14 ingression I (or slow phase) (Fig 2D).

During cycles 10 through 12, the duration of the stabilization phase stays at approximately 3.5 minutes despite the lengthening of the furrow cycles. At cycle 13, stabilization increases to 5.1 minutes (Fig 2A and 2F), however, the duration of stabilization phase remains at ~30% of total cycle time of cycle 13. In contrast, Ingression II doubles its duration at cycle 13 (Fig 2A and 2C). In general, the additional cycle time that occurs with each cycle is largely distributed to the introduction of an Ingression II, with minor contributions to longer stabilization (cycle 13) and Ingression I (cycle 11–13). Thus, an integration of increased duration periods with higher ingression rates directs the lengthening of plasma membrane furrows and produces a rapid change in furrow dimensions in less than 50 minutes of early development.

### Apical budding and furrow displacements

Previous work has shown that an actin-dependent apical budding process begins at cycle 10 when nuclei assume a subcortical position [3, 23]. Given the biphasic furrow dynamics we observed, we wanted to know how apical budding correlates with Ingression I and Ingression II. We therefore measured apical and basal actin displacements (GFP:moeABD) along with furrow ingression (Gap43:mCh plasma membrane marker; Fig 1D). Positive apical displacements, which project outwards towards the extracellular space, are first observable at telophase of the previous cycle (Fig 1D; S1E Fig). This is a period when furrows are either not ingressing (cycle 10) or still retracting from the previous cycle (cycles 11–13) (Fig 1D; S1F Fig). Apical budding is also most pronounced at cycle 10, and apical displacements become sequentially less with successive cycles (S1G Fig). However, budding peaks after ~3 minutes elapsed time, in a period when Ingression I rates also begin to peak. Given that Ingression I also operates early in each cycle, this suggests a possible link between F-actin networks, the apical budding process, and the basal furrow displacements that define Ingression I. To examine this further, we measured the basal extent of F-actin and the furrow tip. Similar to our previous findings, a basal filamentous actin networks extends to approximately 1μm above the furrow tip [5] (Fig 1D).

### MBT regulation of furrow dimensions through zygotic gene expression

**Fig 3:**
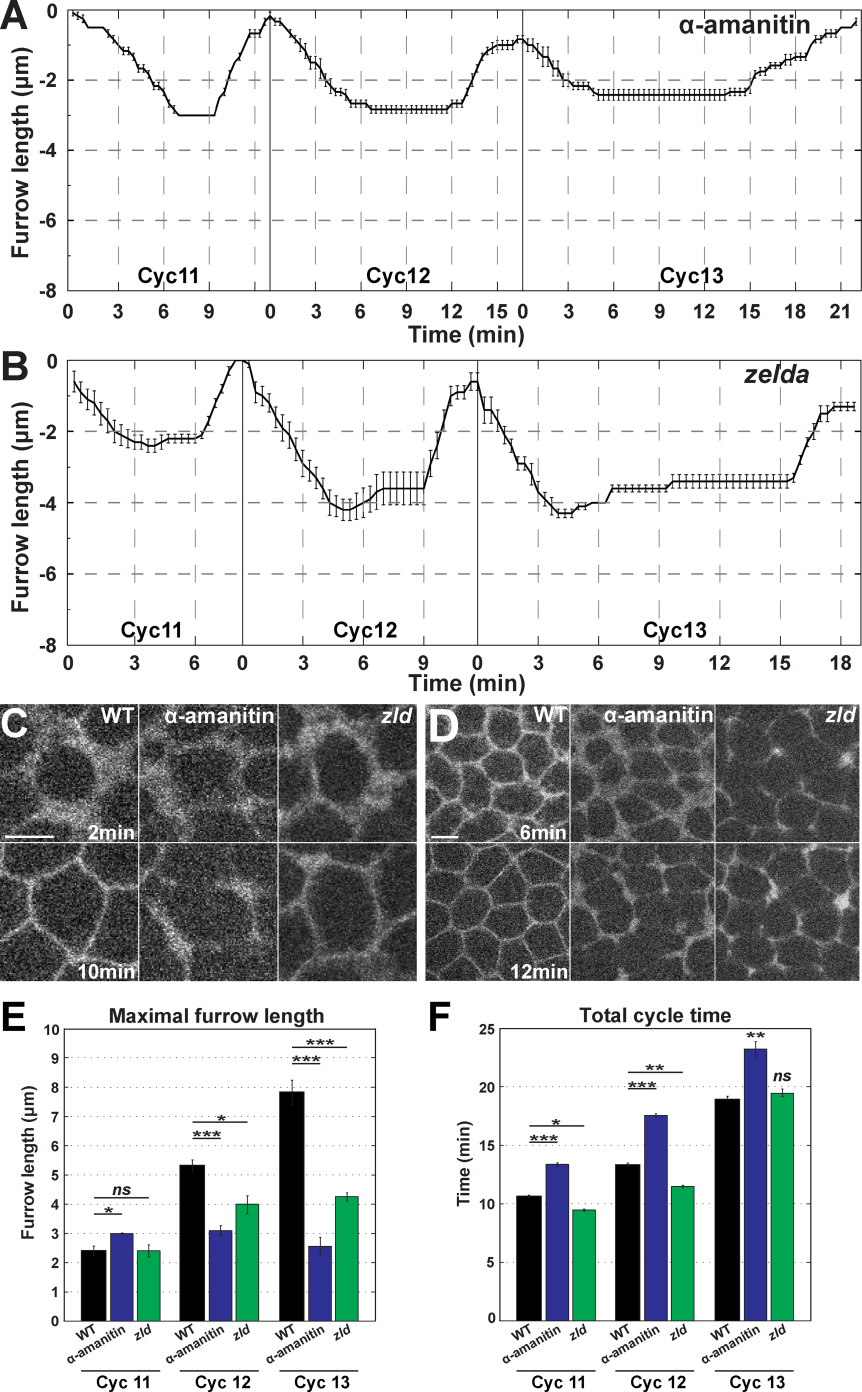
Zygotic gene activation is required for changes in furrow dimensions. (A) Furrow dynamics for α-amanitin injected embryos during cycle 11–13 (cycle 11 and 12: n = 5; cycle 13: n = 4). (B) *zld* mutant furrow dynamics from cycle 11–13 (n = 5). (C) Furrow morphology in WT, α-amanitin injected, and *zld* mutant embryos at 2 min and 10 min in cycle 13. A region just adjacent to the furrow tips is shown. Scale bar = 5 μm (D) Broken furrow phenotype in α-amanitin injected and *zld* mutant embryos at 6 min and 12 min in cycle 13. A region just adjacent to the furrow tips is shown. Scale bar = 5 μm (E) Maximal furrow ingression rates of α-amanitin injected and *zld* mutant embryos from cycle 11–13 (n≥4). *:p<0.05; **: p<0.005; ***: p<0.0005; *ns*: not significant. (F) Total cycle time of α-amanitin injected and *zld* mutant embryos from cycle 11–13 (n≥4). *:p<0.05; **: p<0.005; ***: p<0.0005; *ns*: not significant.

The activation of zygotic gene expression occurs in the same temporal period as the rapid changes in furrow dimensions. To examine if zygotic guidance of furrow behaviors directs furrow morphologies, we injected embryos with α-amanitin, a drug that selectively inhibits RNA polymerase II [24, 25]. Strikingly, blocking zygotic gene expression by treatment with α-amanitin ablates Ingression II (Fig 3A). Furrow formation across syncytial cycles becomes more uniform, and furrows ingress to only 3μm in total. Ingression I, and the initiation of stabilization and retraction phases, appear unaffected, although the period of stabilization is increased by the amount of time that would normally comprise Ingression II. Broader furrow morphologies characteristic of Ingression I are maintained throughout furrow invagination, and small interruptions in furrow continuity become apparent at cycle 13 (Fig 3C and 3D). These data demonstrate the unexpected finding that the zygotic genome is actively regulating furrow behaviors and plasma membrane morphogenesis prior to cycle 14 and the classic definitions of the MBT. The conservation of the temporal period of Ingression II in α-amanitin injected embryos further suggests that furrow dynamics do not drive the initiation of regression, or the termination of stabilization.

We then genetically perturbed the initiation of zygotic gene expression by examining embryos mutant for *zelda*. Regarded as a master regulator of zygotic genome activation (ZGA), *zelda* is a transcription factor required for the expression of a wide portion of early zygotic genes (*zelda*-dependent genes) [14, 16–18, 26, 27]. Similar to α-amanitin injection, in *zelda* mutant embryos Ingression II is almost entirely lost, although furrows proceed ~1μm deeper than in α-amanitin embryos (Fig 3B). In addition, furrow depths during stabilization do not remain at a constant level, and begin to slightly regress after reaching maximum lengths. Furrow lengths remain consistent at 4μm during cycles 12 and 13 (Fig 3E), and Ingression I, Stabilization, and Retraction phases are largely unaffected. Furrow morphologies sharpen slightly more in *zelda* mutants than in α-amanitin embryos, but have similar breaks in continuity (Fig 3C and 3D). The slight deepening of furrows in *zelda* mutant embryos as compared to α-amanitin treated embryos suggests a minor contribution of *zelda*-independent genes to changes in furrow dimensions. *zelda*-independent genes also appear to antagonize factors required for the maintenance of furrow lengths during stabilization phase, as α-amanitin embryos do not display the slow regression of furrows that begins at the end of Ingression I in *zelda* mutant embryos. Alternatively, Zelda mutant embryos possess a slightly deeper Ingression I than wild-type embryos, and the observed regression may represent a reversion to wild-type depths.

A possible simple model to explain changes in furrow dimensions is that as nuclear cycle times increase, this allows a longer period for furrow ingression to occur, thus driving the lengthening of furrows with each successive cycle. However, overall cycle times in α-amanitin and *zelda* mutant embryos are either longer or very similar to wild-type cycle times (WT = 18.8±0.42 min, α-amanitin = 22.2±0.22 min, and *zelda =* 19.0±0.19 min at cycle 13; Fig 3F). These data suggest that zygotic transcripts are directly required in the regulation of furrow lengthening during the syncytial cycles. These results further define maternal and zygotic contributions to furrow behaviors, with Ingression I reliant on maternal gene products, while Ingression II is driven by zygotic gene expression.

### Aneuploid X chromosome embryos reveal zygotic loci that regulate furrow dynamics

**Fig 4:**
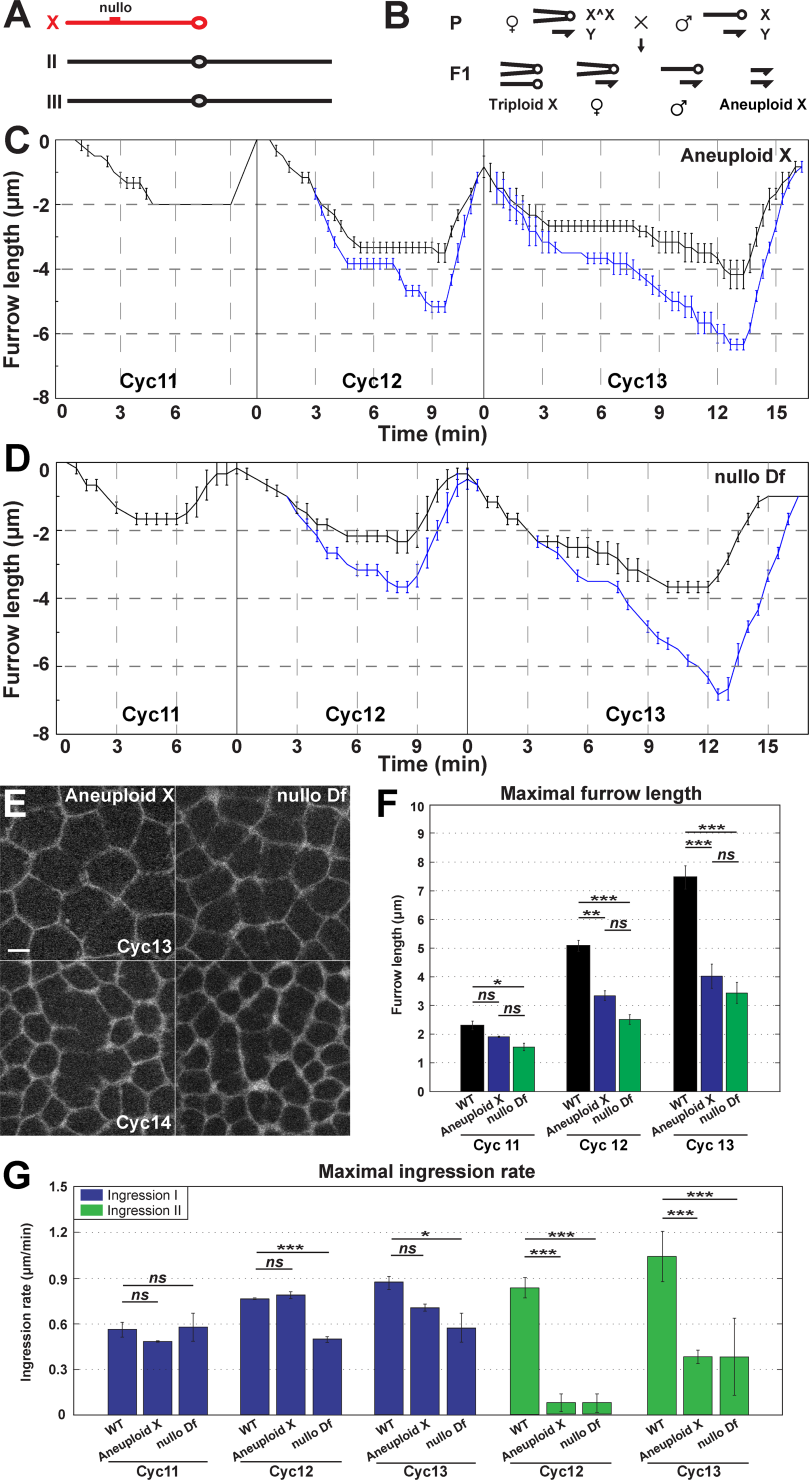
Regulation of furrow dynamics by X chromosome zygotic loci. (A) The major *Drosophila* chromosomes, with the telocentric X chromosome indicated in red. (B) Schematic depicting segregation of compound chromosomes generating X aneuploidy. (C) Aneuploid X chromosome embryo measurements of intact furrow depths (black curve) or deepest extent of fragmented furrows (blue curve) (n = 3). (D) *nullo* Df embryo measurements of intact furrow depths (black curve) or deepest extent of fragmented furrows (blue curve) (n = 3). (E) Disrupted furrow phenotype in aneuploid X and *nullo* Df embryos during cycle 13 and slow phase of cycle 14. A region just adjacent to the furrow tips is shown. Scale bar = 5 μm (F) Maximal furrow length in WT, aneuploid X and *nullo* Df during cycles 11–13 (n≥3). *:p<0.05; **: p<0.005; ***: p<0.0005; *ns*: not significant. (G) Maximal furrow ingression rate of WT, aneuploid X and *nullo* Df during cycles 11–13 (n≥3). *:p<0.05; ***: p<0.0005; *ns*: not significant.

To further investigate the contribution of zygotic chromosomes to the regulation of furrow dynamics, we used compound-X lines to examine embryos that are null for the X chromosome (Fig 4A and 4B). Compound-X stocks were crossed with Gap43:mCh, His2Av:GFP recombinant chromosomes to create embryos expressing markers for the plasma membrane and chromosomes, respectively. Syncytial furrow dynamics were then imaged and analyzed (Fig 4C). In embryos lacking X chromosome function, Ingression II dynamics are deeply compromised, with only a very slight Ingression II retained during cycle 13 (Fig 4C). Furrows ingress to ~4μm during cycles 12 and 13, and maximum ingression rates during Ingression II are greatly reduced, while Ingression I rates are unaffected (Fig 4F and 4G). These results support that zygotic loci direct changes in furrow dimensions, and suggest that X chromosomal genes are essential drivers of Ingression II.

X chromosome deficient embryos also possess further changes in furrow morphologies. Compound-X embryos show a “broken furrow” phenotype by cycle 13 that is similar to what is observed in α-amanitin and *zelda* embryos (Fig 4E; S2A Fig). Furrow lengths are 1–2μm deeper when the extent of these fragmented furrows is measured (blue line, Fig 4C; S2B Fig). These broken furrow defects are also reminiscent of those observed in *nullo* mutant embryos during cellularization [28–30]. We performed immunostaining for Nullo protein and found it is present prior to cycle 14 [31] (S2C Fig). As *nullo* is located on the X chromosome, we examined *nullo* deficient embryos to see if a canonical cellularization and MBT-associated gene is required during earlier cycles (Fig 4D). Indeed, *nullo* mutant embryos display defective furrow morphologies prior to cellularization (Fig 4E). Additionally, *nullo* mutant embryos possessed shortened furrows, decreased ingression rates, and defects in Ingression II, demonstrating that *nullo* is likely the predominant locus on the X chromosome regulating changes in furrow lengths (Fig 4D–4G). However, similar to X chromosome deficient embryos, furrow lengths are several microns longer if the deepest extent of fragmented furrows is measured (blue line, Fig 4D; S2B Fig).

### Autosomal contributions to furrow regulation

**Fig 5:**
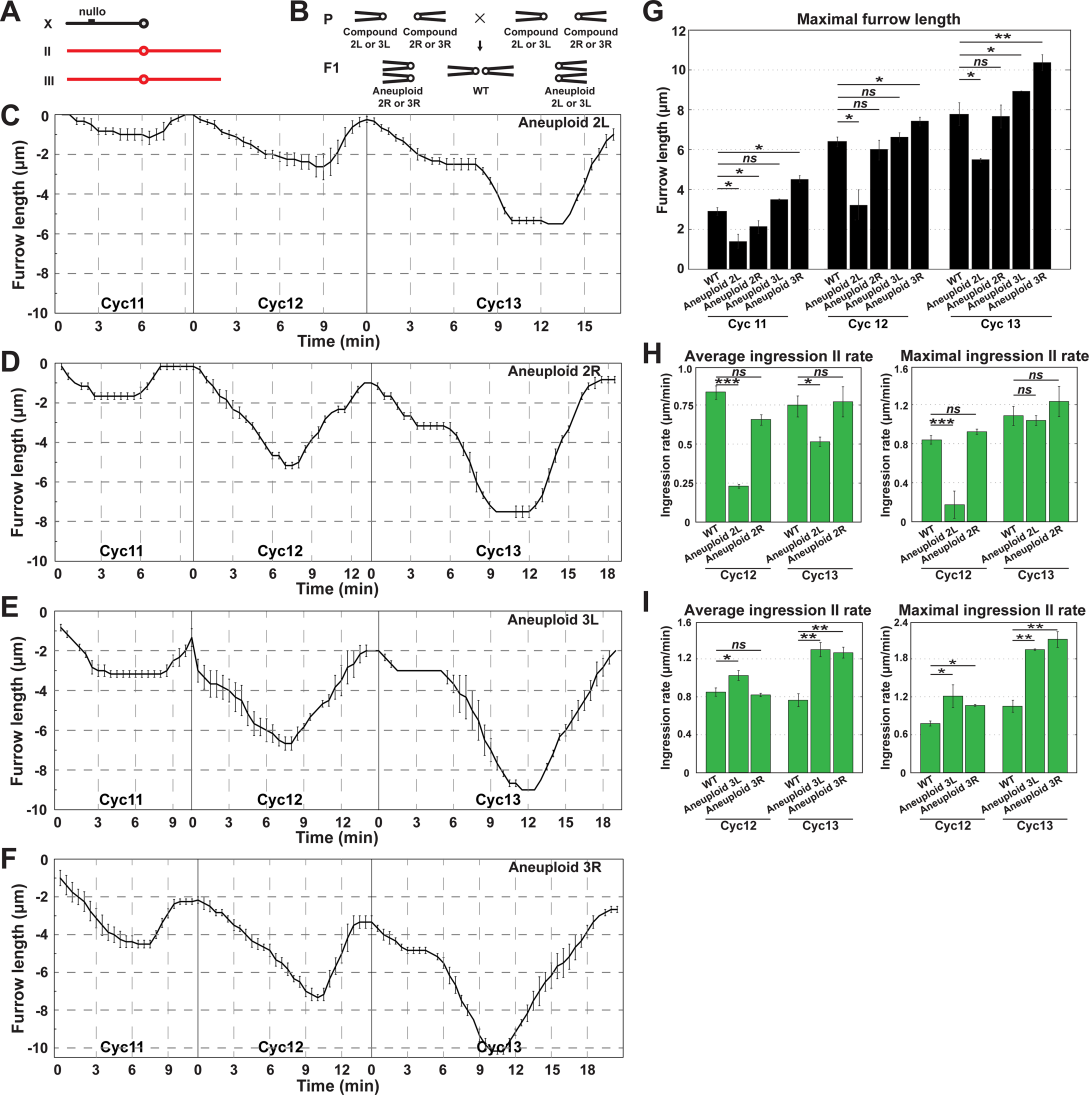
Autosomal contributions to furrow regulation. (A) The major *Drosophila* chromosomes, with the metacentric autosomes indicated in red. (B) Schematic depicting segregation of compound chromosomes generating 2L, 2R, 3L or 3R aneuploidy. (C) Aneuploid 2L furrow dynamics (n = 3). (D) Aneuploid 2R furrow dynamics (n = 4). (E) Aneuploid 3L furrow dynamics (n = 3). (F) Aneuploid 3R furrow dynamics (n≥3). (G) Maximal furrow length of WT, aneuploid 2L, 2R, 3L and 3R during cycles 11–13 (n≥3). *:p<0.05; **: p<0.005; ***: p<0.0005; *ns*: not significant. (H) Average and maximal Ingression II rates of WT, aneuploid 2L and 2R embryos during cycles 11–13 (n≥3). *:p<0.05; ***: p<0.0005; *ns*: not significant. (I) Average and maximal Ingression II rates of WT, aneuploid 3L and 3R embryos during cycles 11–13 (n≥3). *:p<0.05; **: p<0.005; ***: p<0.0005; *ns*: not significant.

Given that disrupting X chromosomal function led to the identification of zygotic factors required for furrow stability and lengthening, we then examined the contributions of chromosomes II and III, the major autosomal chromosomes of *Drosophila melanogaster*. We imaged embryos from compound II and compound III stocks that generate aneuploidy for either the left or right arms of chromosomes II and III (2L^-^, 2R^-^, 3L^-^, and 3R^-^; Fig 5). This analysis revealed that a major locus required for furrow lengthening is apparent on the left arm of chromosome II, while 2R^-^ embryos possessed largely wild-type furrow dynamics (Fig 5C, 5D and 5G). Ingression II rates are deeply reduced in 2L^-^ embryos, but not significantly changed in 2R^-^ (Fig 5H). Interestingly, aneuploidy for either 3L or 3R drove a slight *deepening* of furrows in each of these genetic backgrounds. Indeed, average and maximal Ingression II rates were increased during cycle 13, suggesting that factors that antagonize furrow invagination are present on chromosome III (Fig 5E–5I). Autosomal aneuploidies had minor effects on Ingression I rates, although 2L^-^ embryos had an ~50% reduction in average Ingression I rates (S3A and S3B Fig). These results demonstrate that zygotic loci on the X and autosomal chromosomes drive changes in furrow dimensions and function in development prior to cycle 14.

### Correspondence of furrow dynamics to the cell cycle

**Fig 6:**
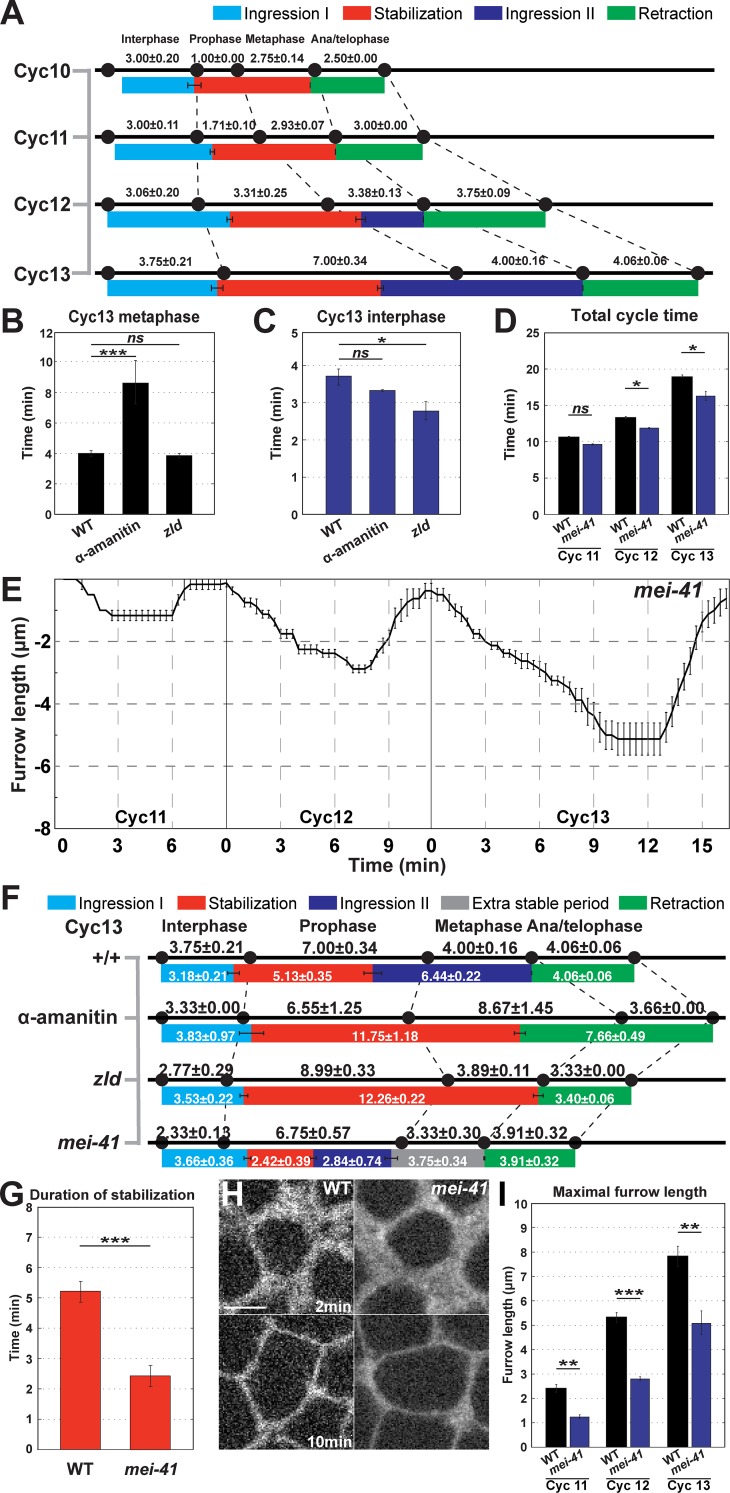
The cell cycle is a permissive cue for furrow dynamics. (A) Phases of WT furrow dynamics and their correlation to cell cycles during cycles 10–13 (n≥4). (B) Cycle 13 metaphase duration in WT, α-amanitin injected, and *zld* mutant embryos (n≥4). ***: p<0.0005; *ns*: not significant. (C) Cycle 13 interphase duration in WT, α-amanitin injected, and *zld* mutant embryos (n≥4). *:p<0.05; *ns*: not significant. (D) Total cycle time of WT and *mei41* mutant embryos during cycles 11–13 (n≥3). *:p<0.05; *ns*: not significant. (E) *mei41* mutant furrow dynamics (cycle 11:n = 3; cycle 12 and 13: n = 4). (F) Correspondence of furrow phases and the cell cycle in WT, α-amanitin injected, *zld*, and *mei41* mutant embryos during cycle 13 (n≥3). (G) Duration of stabilization phase of WT and *mei41* mutant embryos during cycles 11–13 (n≥3). ***: p<0.0005. (H) Furrow morphology in WT and *mei41* mutant embryos, at 2 min and 10 min in cycle 13. A region just adjacent to the furrow tips is shown. Scale bar = 5 μm (I) Maximal furrow ingression rate of WT and *mei41* mutant embryos during cycles 11–13 (n≥3). **: p<0.005; ***: p<0.0005.

To examine the relationship between furrow dynamics and the cell cycle, we analyzed the correspondence between chromosomal behaviors and Ingression I, Stabilization, Ingression II, and Retraction. By tracking changes in Histone:GFP-marked chromosomes, we were able to define interphase periods, as well as the relative timings of prophase, metaphase, and anaphase. We find that Ingression I initiates at the beginning of a new cell cycle, even as furrows have not fully retracted back to the apical surface (Fig 1B and 1C; Fig 6A). Ingression I proceeds for the next 3–4 min, before Stabilization initiates as embryos enter into prophase and the first signs of chromatin condensation are visible (Fig 6A; S3C Fig). Stabilization initially corresponds to the periods when prophase and metaphase occur. However, with the initiation of Ingression II in cycles 12 and 13, the correspondence between the end of stabilization and the cell cycle begins to erode (Fig 6A). Consistent with this, Ingression II begins near the start of metaphase in cycle 12, but then initiates during prophase of cycle 13. Ingression II terminates at anaphase in both cycles 12 and 13, followed by furrow retraction. This correspondence of furrow retraction with anaphase occurs throughout the syncytial cycles.

We also analyzed how cell cycle dynamics changed in various compromised backgrounds. As described above, α-amanitin injection lengthens overall cell cycle times, although this does not lead to longer furrow lengths. It is interesting to note that much of the increase in cell cycle time goes into an elongation of the time of metaphase (Fig 6B*)*. However, despite this elongation of metaphase, Ingression II is still absent. Similarly, *zelda* mutant embryos have a shortened interphase, but possess a deeper Ingression I (Fig 6C). These data demonstrate that, while there is a partial correspondence between furrow behaviors and markers of cell cycle progression, the phases of the cell cycle are not strictly deterministic in the regulation of furrow dimensions.

### Checkpoint function is required for stabilization and full expression of ingression II

While lengthening cell cycle times does not lead to the deepening of furrow dimensions in the absence of zygotic transcription, we examined if checkpoint function is required to permit the expression of zygotic gene products and subsequent furrow regulation. Mei41 functions as a checkpoint protein that, when mutated, leads to shortened cell cycle times and an eventual catastrophic defect in genomic stability at cycle 14 [13, 32–35]. In maternally mutant *mei41* embryos, the overall cycle times display no significant difference prior to cycle 12 (Fig 6D and 6E). However, at cycle 12 checkpoint function is required to initiate a wild-type cycle time (Fig 6D and 6E). Interestingly, *mei41* checkpoint function is also essential for the full ingression of syncytial furrows and for triggering the Stabilization phase (Fig 6E–6G). In the absence of checkpoint function, Stabilization becomes unstable at cycle 12, and by cycle 13 is deeply compromised (Fig 6E–6G). During cycle 13, furrow depth begins to plateau after Ingression I, but then ingression rates accelerate again and a smooth transition to a short Ingression II occurs (Fig 6E and 6F; S3D Fig). Furrow tip morphologies successfully transition from broader Ingression I furrows to the sharper, more defined morphologies characteristic of Ingression II (Fig 6H). However, furrow depths reach only 5μm, and do not reach wild-type depths (Fig 6I). Maximum furrow depths are also reached earlier in the cell cycle (at 8.9 minutes in *mei41* mutants versus 13.7 minutes in wild-type cycle 13 embryos), and then appear incapable of further ingression (Fig 6E and 6F). As *mei41*, *zelda* double mutant embryos have been reported to suppress mitotic catastrophe in the early embryo [13], we also examined embryos with compromised *mei41* and *zelda* function to see if there is a similar rescue of furrow ingression. However, furrow ingression depths and rates are still deeply compromised in *mei41*, *zelda* defective embryos (S3E–S3G Fig). These results suggest that cell cycle checkpoint function is necessary to permit the full function of zygotic gene products in directing changes in furrow dimensions, and further reveal that checkpoint function is necessary for the normal initiation of the Stabilization phase.

### Maternal transcript clearance is a minor contributor to furrow regulation

**Fig 7:**
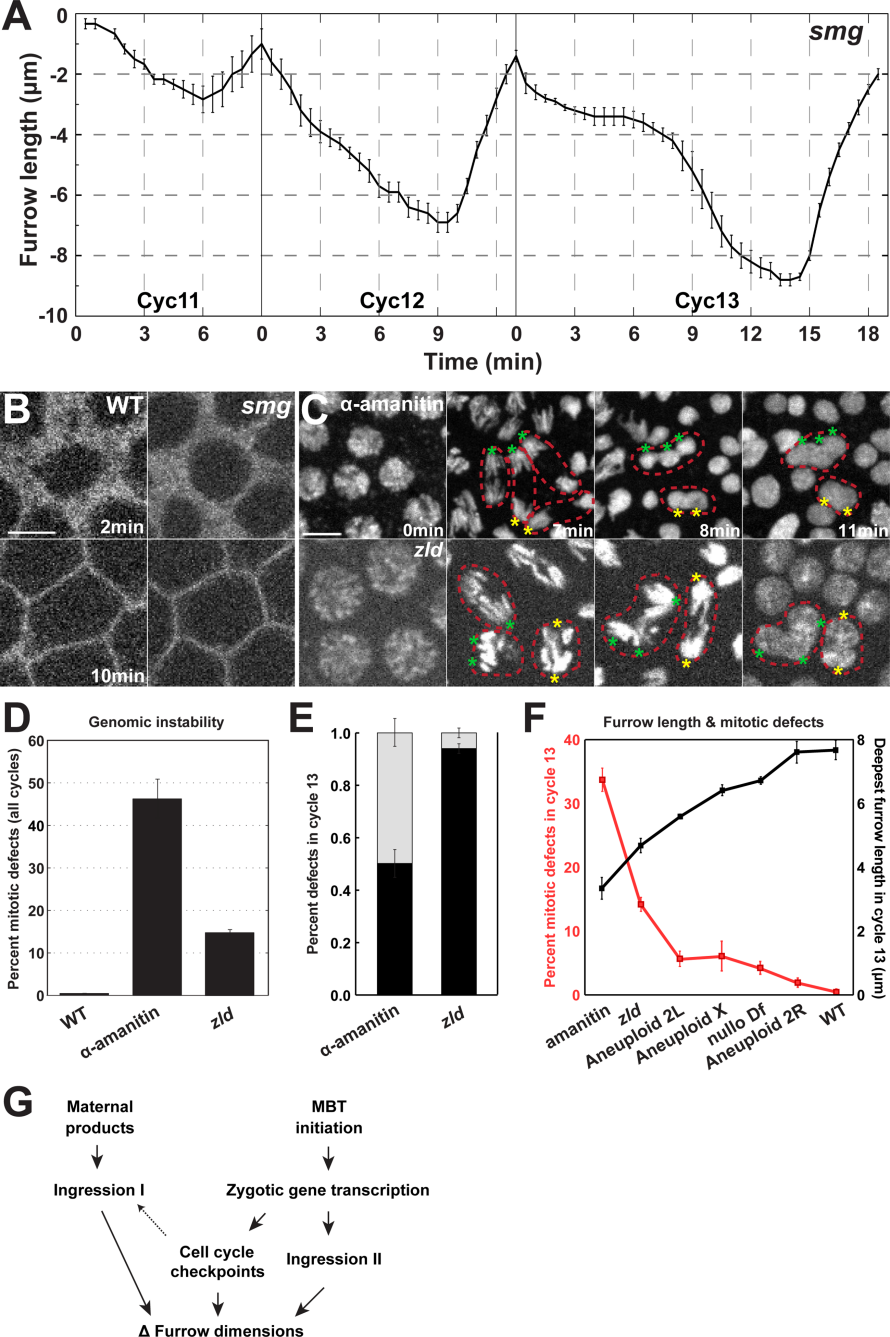
Maternal gene decay is a minor contributor to furrow dynamics. (A) *smg* mutant furrow dynamics (cycle 11: n = 3; cycle 12 and 13: n = 5). (B) Furrow morphology in WT and *smg* mutant embryos at 2 min and 10 min. A region just adjacent to the furrow tips is shown. Scale bar = 5 μm (C) Mitotic defects in α-amanitin injected and *zld* mutant embryos. Chromosomes are labeled with Histone:GFP. Red, dotted lines highlight individual mitotic figures that fail to properly segregate chromosomes resulting in polyploid nuclei, and asterisks indicate missegregating chromosomal complements. (D) Percent of nuclei that experience mitotic defects in WT, α-amanitin injected, and *zld* mutant embryos. (E) Percent of adjacent nuclear fusion (black bar) and mitotic nuclear fusion (grey bar) in α-amanitin injected and *zld* embryos (n = 3) in cycle 13. (F) Furrow length and mitotic defects are inversely correlated. The percentage of mitotic defects at cycle 13 in α-amanitin injected, *zld*, Aneuploid 2L, Aneuploid X, *nullo*, Aneuploid 2R, and WT embryos is presented, as well as deepest furrow lengths during metaphase in cycle 13. Intact furrow length and corresponding mitotic defects are measured in S3J Fig (G) Model for developmental regulation of furrow dimensions in the early *Drosophila* embryo.

In addition to testing the function of zygotic genome activation in the MBT-driven regulation of furrow behaviors, we also characterized the contribution of the other major contributor to the MBT–the decay of maternal gene products. Smaug (smg) is an essential factor for maternal mRNA destabilization [19, 20]. While additional transcript clearance pathways exist (for example, *BRAT* and *pumilio* dependent pathways), *smg* appears to have the earliest function in maternal clearance [19, 36]. We therefore used *smg* mutant alleles to examine the effects of maternal gene decay on furrow dynamics. *smg* mutant embryos do not show gross disruptions of furrow behaviors of the kind observed in α-amanitin, *zelda*, compound-X, or *mei41* compromised embryos. *smg* embryos have similar furrow behaviors and display dynamics comparable to wild-type embryos (Fig 7A and 7B). However, furrows are ~1μm deeper in each cycle than in wild-type. This *smg*-dependent effect on furrow lengths does not change dynamically during the syncytial cycles. Thus, smg-dependent maternal gene decay has a small effect on furrow depths and appears to be a minor contributor to furrow behaviors in the early embryo. These results are also consistent with previous studies that show that the major portion of Smg-induced maternal mRNA destabilization occurs at cycle 14 [11,19,21].

### Furrow lengthening is essential to genomic stability and chromosomal segregation

The deepening of furrows occurs in a stepwise fashion with each round of nuclear division, and raises the question of what are the functional consequences of furrow lengthening. In each round of nuclear divisions, the number of nuclei located in a common subcortical plane will double. Indeed, nuclear densities increase from 2.3 nuclei/1000μm^2^ at cycle 10 to 15.4 nuclei/1000μm^2^ at cycle 13 (S3K Fig), and 29.8 nuclei/1000μm^2^ at cycle 14. This crowding together of nuclei suggests that a greater separation between nuclei may be necessary to maintain genomic stability. Previous work has demonstrated that, in the absence of furrow formation, the separation of nuclei fails and polyploidy occurs through the fusion of chromosomal complements during mitosis [3–5]. However, whether furrow lengthening is essential to the maintenance of genomic stability has not been addressed. We therefore examined α-amanitin and *zelda* mutant embryos in which furrow lengths remain relatively constant and Ingression II does not occur. Indeed, in these backgrounds, the importance of increasing furrow dimensions is apparent. By the end of cycle 13, genomic stability has become deeply compromised, with 47.1% (α-amanitin) and 16.2% (*zelda*) of nuclei becoming polyploid through fusion events and/or failures in chromosomal segregation (Fig 7C–7E, S3H Fig). By following individual mitotic figures, it became clear that mitotic defects arise through either a failure to separate adjacent mitoses or through a collapse of individual mitotic figures (S3I Fig). Similar defects are also seen in compound II mutant embryos in which furrow lengths are decreased, and an inverse relationship between furrow length and chromosomal missegregation is apparent (Fig 7F). It is interesting to note, however, that embryos with compromised X chromosome function display a low level of genomic instability (compound X = 7.6%, *nullo* Df = 4.5% at cycle 13, Fig 7F), consistent with previous reports [28, 29]. This suggests that the partial furrows that can extend, with breaks in their continuity, to ~7μm are sufficient to provide the separation functions that ensure appropriate mitotic divisions (Fig 7F, S2A Fig). These results demonstrate the essential requirement for MBT-regulated changes in furrow dimensions in the maintenance of normal chromosomal segregation in the early embryo.

## Discussion

The ability to form a plasma membrane furrow is essential to most cellular and tissue-level developmental processes, and plasma membrane furrow formation is fundamental to successful cytokineses. Here, we have shown that furrow lengthening occurs through a biphasic process. The initiation of syncytial furrow formation at cycle 10 occurs through a primary ingression phase that smoothly transitions into a stable period, before furrows retract back to the apical surface. However, as development proceeds, furrow depths will gradually lengthen, finally extending more than 18-fold deeper during cellularization of cycle 14 as compared to initial furrows at cycle 10. This change in furrow dimensions is driven by the coordination of several factors (Fig 7G). Cell cycle times increase with each round of division, which permits modest increases in ingression periods for Ingression I. However, the major change in furrow dimensions is the result of the introduction of a second ingression phase, Ingression II. Ingression II is capable of higher maximum rates, proceeding as much as 4x faster than Ingression I. This higher ingression rate, combined with an extended cycle time, permits the formation of deep furrows required for genomic stability and the anchoring and segregation of mitotic spindles in later cycles. These results further show that furrow lengthening is not driven solely by the prolonged maintenance of initial processes, but is aided by the addition of an active, more rapid, pathway.

### Developmental control of plasma membrane furrow invagination

Changes in furrow dimensions occur during the same period as activation of the zygotic genome and the degradation of maternally-deposited gene products. Blocking zygotic transcription through α-amanitin injection leads to a loss of furrow deepening, and the disruption of Ingression II. However, Ingression I still occurs in a largely wild-type fashion in α-amanitin treated embryos. This suggests that Ingression I is primarily driven by maternal protein products, while Ingression II is directed by factors derived from the zygotic genome. Activation of the zygotic genome has been shown to occur in a stepwise fashion, through the regulation of chromatin states and the accessibility of enhancer and promoter elements [18]. The earliest zygotic pool of coordinately regulated genes is activated through the functioning of the Zelda transcription factor [14, 15, 17, 18]. Disruption of *zelda* function led to furrow phenotypes that were similar to α-amanitin injection. Furrows extended slightly deeper in *zelda* mutant embryos, suggesting a minor contribution to Ingression II from *zelda*-independent genes. It was also interesting that as furrows reached a maximum depth in cycles 12 and 13, they immediately began a slow regression, suggesting that zygotic gene products are required for furrow maintenance. One future direction will be to explore the nature of this furrow maintenance. By contrast, the turnover of maternal products did not appear to play a major role in driving changes in furrow dimensions. *Smg* mutant embryos showed largely wild-type dynamics, although furrows extended slightly deeper with each cycle. This potentially suggests that the clearance of maternal products mildly restrains furrow lengthening during the syncytial cycles. However, it may be that additional degradation pathways exist in the early cycles that, when disrupted, will demonstrate deeper effects on furrow morphologies.

### Aneuploid chromosomal approaches identify zygotic contributions

The use of compound chromosomal stocks permits the rapid identification of genomic contributions to early developmental processes [28, 37, 38]. Compound chromosomal stocks also have the advantage of not possessing maternal heterozygosities, as is the case for most smaller deficiencies and alleles. This permits the examination of purely zygotic contributions to early furrowing events. Utilizing this approach, we observed that major loci on the X and 2L chromosomes controlled furrow ingression dynamics. Minor contributions from 3L and 3R were also observed. It should be noted that autosomal compound stocks generate aneuploidies for given chromosomal arms, but that these embryos are also tetraploid for the opposing chromosomal arm (see genetic schema in Fig 5B), raising the formal possibility that furrow phenotypes could be driven by extra copies of zygotic gene products. However, the broken furrow phenotype from compound X embryos suggested that a loss of *nullo* function, which is located on the X chromosome, could contribute to furrow ingression prior to cycle 14. Indeed, a small deficiency uncovering the *nullo* gene reproduced much of the aneuploid X phenotypes and lacked Ingression II. This is intriguing, as *nullo* has been a classic example of a cycle 14 MBT gene, and further suggested a potential homology between the early syncytial furrows of cycles 10–13 and cellularization at cycle 14. However, it is interesting to note that previous work has suggested that ingression rates in *nullo* mutant embryos during cellularization are close to wild-type levels [31, 39]. Similarly, if the deepest extent of broken furrows is tracked in our data, there is only a 1–2μm difference between *nullo* mutant and wild-type embryos.

### The nature of the MBT and morphogenesis

The transition between maternally-driven morphogenesis to the guidance of the embryo’s own genome is an event that must occur in almost all higher organisms [40–42]. In *Drosophila*, the MBT has classically been considered to occur at cycle 14. This has also corresponded with one of the major morphogenetic events in the early embryo, the formation of a monolayered epithelial sheet through the process of cellularization. Here, we have shown that many, although not all, of the furrowing dynamics required for cellularization are established during the earlier syncytial divisions. It is interesting to note that several recent studies on the initiation of zygotic transcription have also indicated a broader temporal start to these events [10–13]. Indeed, a few examples of zygotically driven morphogenesis prior to cycle 14 have been previously reported and suggest developmental roles for early gene transcription. Different domains of anterior nuclear densities pre-pattern cellularization and are generated during cycles 10–14 [43]. Additionally, *engrailed*-dependent positioning of the pole cells occurs at cycle 10 in response to an early-driven transcript [9,44]. These results are inconsistent with a sharp cycle 14 midblastula transition, and instead suggest a gradual transition to zygotically-driven development and morphogenesis.

### A permissive, rather than instructive, role for the cell cycle in early furrow dynamics

Many higher organisms, including *Drosophila*, start development with abbreviated cell cycles that rapidly alternate between S and M phases [45–47]. As these early cell cycles proceed, the duration of cell cycles slowly extends until embryos reach the MBT. Recent work in *Drosophila* has shown that cell cycle checkpoints become engaged as transcription from the embryo’s own genome begins, and that checkpoint function is required for the full production of transcript products during cycle 12–14 [13]. Our work has demonstrated that these zygotic products are essential to the lengthening of furrow dimensions. We also have shown that Mei41 checkpoint function is needed for wild-type furrow production. It is interesting to note, however, that although furrow lengths are reduced in *mei41* mutant embryos, Ingression I, Stabilization, and Ingression II periods still occur at cycle 13. Ingression II, Stabilization, and overall cell cycle times are greatly reduced, though. This data would be consistent with two possible interpretations of Mei41 checkpoint function: 1) a direct model, in which shorter cell cycle times limit the periods that allow furrow ingression, or 2) an indirect model, in which the failure of checkpoint function disrupts the amount of zygotic transcription that can occur. As furrows in *mei41* mutants plateau several minutes before anaphase and the usual termination of Ingression II, we favor the second model in which products essential to furrow ingression are not made at high enough levels to support a continued furrow ingression during metaphase of cycle 13. This would also be consistent with data from α-amanitin treated embryos in which the cell cycle is longer than in wild-type embryos, but furrow lengths are shortened. Additionally, although furrow processes often have a general correspondence to phases of the cell cycle, in various mutant and small molecule-treated backgrounds these associations break down. These results suggest that cell cycle elements that regulate interphase and mitotic periods have a permissive, rather than strictly deterministic, effect on furrow dynamics.

### Early furrow function and genomic stability

Furrows progress from 1.5μm at cycle 10 to 8μm at cycle 13 and 28μm at cycle 14. This deepening of furrows is associated with an increase in nuclei number, and suggests a functional role for furrows in maintaining proper chromosomal segregation. Indeed, throughout the early cell cycles there is an inverse relationship between furrow length and mitotic defects. This mirrors data for furrow-less embryos, in which chromosomal segregation defects are apparent from cycle 11 through cycle 13, although cycle 10 embryos are relatively free of mitotic defects [5]. At cycle 13, when mitotic nuclei are most densely packed, there appears to be a critical threshold in furrow length at ~4μm for genomic stability. If furrow lengths are shorter than 4μm, a near majority of nuclei will experience mitotic defects. However, even a relatively small, 2μm reduction in furrow lengths at cycle 13 will produce a low level of polyploid nuclei (5%).

Live imaging of chromosomal dynamics also permitted the tracking of individual mitoses, and demonstrated that defects in segregation occur through two possible events: 1) the fusion of adjacent chromosomal complements, usually after the successful completion of anaphase, or 2) the collapse of individual mitotic figures [5] (Fig 7C; S3I Fig). The fusion of adjacent chromosomes appears to be a direct consequence of defects in furrow ingression, as chromosomal complements are not separated during the latter stages of mitoses and become packaged into common, polyploid nuclei. In *zelda* mutant embryos, ~95% of segregation defects are through adjacent chromosomal fusions. However, the mitotic collapse phenotype could be the result of either a lack of furrows to properly anchor and attach the mitotic spindle, or could be a result of cell cycle defects that cause tangled chromosomal complements that inhibit separation. Previous work on furrow-less embryos generated by defects in membrane trafficking has shown that both the adjacent fusion and mitotic collapse phenotypes occur in embryos that still possess otherwise wild-type cell cycle times and behaviors [5]. This does not rule out a role for cell cycle dysregulation in driving chromosomal segregation defects, although as segregation defects are observed in a variety of different backgrounds and pathways that compromise furrow behaviors (defects in membrane trafficking, zygotic transcription, aneuploid chromosomes, and cell cycle checkpoints) this seems less likely. It is interesting to note that genomic instabilities such as these are common in many forms of human cancers [48]. It also appears that many cancers have early initiating events that can result from failures in cytokinetic furrows [49, 50]. As complex tissues contain cells in a variety of different shapes and sizes, it will be intriguing to explore if early oncogenic events may be due to failures in maintaining proper cytokinetic furrow length that then consequently leads to genomic defects.

## Materials and methods

### Fly stock and genetics

All fly stocks were maintained at 25^o^ C. The following stocks were used in this study: Gap43:mCherry (plasma membrane and furrow marker, A. Martin, MIT), P{OvoD1-18}, P{neoFrt19A}/C(1)DX/Y; P{hs-Flp} (N. Tolwinski, Yale NUS College, Singapore), and Sqh-GFP:moeABD (the actin-binding domain of moesin fused to GFP, D. Kiehart, University of North Carolina). Resille:GFP (plasma membrane and furrow marker), Spider:GFP (plasma membrane and furrow marker), His2Av:GFP and His2Av:RFP (chromosomal marker), *Df(1)Sxl-bt* (*nullo* Df)/FM7 (Simpson and Wieschaus, 1990), *smg*^1^/TM3 (Dahanukar et al., 1999), and *Df(3L)Scf*^*R6*^/TM3 (Dahanukar et al., 1999) were provided by the Bloomington *Drosophila* Stock Center. *zld*^294^ P{neoFRT19A} (Liang et al., 2008), *mei41*^D3^, *mei41*^29D^ (Banga et al., 1986; Banga et al., 1995), C(1)DX, C(2)v, and C(3)Se were kindly provided by the Wieschaus lab (Princeton University).

*zelda* germline clones were generated by crossing *zld*^294^ FRT19A females to ovoD, FRT19A males. Larvae were heat shocked three times for 2 hours over the course of 5 days to induce recombination events.

To image furrow dynamics in aneuploid backgrounds, Compound X females were crossed to males carrying both membrane and histone markers. To create a Compound II stock carrying both membrane and histone markers, we took advantage of the fact that autosomal, double balancer stocks (CyO; TM3) have a low rate of missegregation defects that generate aneuploid gametes. Sp/CyO; His2Av:RFP, Spider:GFP/TM3 females were crossed to Compound II C(2)v males. Rare F1 males (C(2)v; His2Av:RFP, Spider:GFP/+) were backcrossed to compound stocks and embryos from the F2 progeny were used for imaging. Aneuploid X, 2L or 2R embryos were determined by scoring for phenotypes caused by deficiency of *nullo*, *halo*, or *twist/eve/Kr*, respectively. For compound III analysis, furrow dynamics were followed by extracellular dextran488 injection. Aneuploid 3L and 3R embryos were determined by *fuzzy-cellularization*, and *Serendipity-α*, respectively.

### Microscopy and time-lapse imaging

A spinning-disk confocal microscope from Zeiss/Solamere Technologies Group with 63X/1.4NA objective lens was used for time-lapse imaging. The embryos were collected on standard yeasted apple juice agarose plates, dechorionated, and transferred to an air-permeable membrane in Halocarbon 27 oil (Sigma). A coverslip was placed on embryos for live imaging. For individual time-lapse imaging, full z-stacks were acquired at either 20s or 30s intervals. Each z-stack was comprised of 30–33 z-slices at a 0.5μm interval. For cytoplasmic budding imaging, 30 z-slices with a 0.3 μm interval were taken at every 20s. All movies were acquired at 25°C.

### Embryo fixation, immunostaining and imaging

Embryos were dechorionated in 50% bleach solution and fixed for 20 minutes at the interphase of heptane and either 18.5% formaldehyde (Electron Microscopy Sciences) (for anti-Nullo staining), or 4% formaldehyde (for anti-Lamin staining) in 0.1 M sodium phosphate buffer (pH 7.4). Then the embryos were manually devitellinized and stained with Alexa 546-phalloidin (1:200, Molecular Probes), mouse anti-Lamin (1:1, ADL195, DSHB), or mouse anti-Nullo (1:15, Nullo 5C3-12, DSHB). Secondary antibodies conjugated with Alexa 488 (Molecular Probes) were used at 1:500. Embryos were mounted in ProLong Gold with DAPI staining (Life Technologies). Immunostained embryos were imaged with an Olympus Fluoview FV100 confocal laser scanning microscope with 40X or 60X 1.35NA objective lens. Images were acquired using 12 ms/pixel exposure settings.

### Drug injection

Embryos were glued on a coverslip after dechorionation. The embryos were dehydrated for 12–15 min, covered in Halocarbon oil 700 (Sigma), and injected with α-amanitin (100mM in water, Santa Cruz), Dextran-Alexa488 (1mg/mL; Life Technologies), or water. After injection, the embryos were placed on an air-permeable membrane and imaged on the spinning disk confocal microscope.

### siRNA preparation and injection

Primers for siRNA treatments were designed using the SNAPDRAGON RNAi design program (http://www.flyrnai.org/snapdragon) to decrease potential off-target effects. dsRNA was made using Mega script T7 Transcription Kit (Ambion) and purified by Quick-RNA Microprep kit (Zymo Research). The concentration of dsRNA was determined by a NanoDrop ND1000 spectrophotometer (2000 ng/μL). For injection of siRNA, the embryos were prepared in the same method as drug injection. After injection, embryos on a coverslip were immersed in Halocarbon 27 oil (Sigma) and placed on a gas-permeable membrane and imaged on the spinning disk confocal microscope.

### Furrow dynamics and cycle time measurements

Furrow dynamics and cycle time were measured by live-imaging embryos with both membrane and histone markers. The first, apical z-layer of the furrow was determined as the point at which the apical membranes meet and come to a common width. Furrow ingression was tracked by determining the first moment that intact furrow rings comprising a 4–5 “cell” region had advanced to a new basal layer. For aneuploidy X and null Df with “broken” furrows, the deepest extent was determined by the deepest layer where a partial furrow presented (S2A Fig). Cell cycle status was determined by DNA morphology. Interphase was defined as occurring from the appearance of new nuclei formation to the first appearance of chromosomal condensation, which was indicated by the bright puncta of Histone:GFP. The period between chromosome condensation and nuclear disbandment was defined as prophase, and metaphase was the period from nuclear disassembly to the onset of chromosomal segregation. Anaphase/telophase was determined by the period from chromosomal segregation to the formation of new daughter nuclei. The embryo furrow dynamics was aligned by the onset of segregation. The total time of compound III embryo furrow dynamics was normalized to control water-injected embryos to correct for effects of injection and preparation for injection. WT, α-amanitin injected, *nullo* Df, and compound X furrow dynamics were imaged with Gap43:mCh, while compound II, z*ld* and *mei41* mutant embryos were imaged with Spider:GFP. *smg* mutant embryos were imaged with Resille:GFP, and compound III embryos were imaged by extracellular dextran488 injection. A comparison of furrow dynamics from Gap43:mCh, Spider:GFP, Resille:GFP, and dextran-injection is presented in S1D Fig.

### Mitotic defects measurements

The mitotic defects were measured by live-imaging embryos with both membrane and histone markers. The ratio of mitotic defects was calculated by dividing the number of defective mitoses by the total number of division events in cycle 13. The ratio of fusion nuclei was calculated by dividing the number of fused nuclei by the total number of nuclei in cycle 14. The adjacent nuclear fusion and mitotic nuclear fusion events in cycle 13 were tracked, respectively, and the ratio was calculated.

### Statistics and repeatability

Furrow length, durations, ingression rates, and cell cycle time data were tested for statistical significance using Student’s t-test. *ns*: p>0.05; *:p<0.05; **: p<0.005; ***: p<0.0005.

All measurements were quantified from a minimum of 3 embryos, and represented at least two individual trials.

### Image editing and figure preparation

Spinning disk and laser scanning confocal microscopy images were edited using ImageJ and Adobe Photoshop. Images were uniformly leveled for optimal channel appearance. Furrow dynamics curves were made in OriginLab. Figures were prepared and labeled in Adobe Illustrator.

## Supporting information

S1 Fig Furrow dimensions and morphologies. (A) Wild-type furrow dynamics, including cellularization, during cycles 10–14 (cycle 10: n = 4; cycle 11: n = 7; cycle 12 and 13: n = 8; cycle 14: n = 3). (B) Furrow morphology during cycles 10–13. Scale bar = 5 μm. (C) Wild type furrow dynamics from cycle 12. Red line shows the furrow dynamics without stabilization phase (n = 5 embryos); and black line shows with stabilization phase (n = 7 embryos). (D) Cycle 13 furrow dynamics for different membrane markers and injection controls (n≥3). (E) Apical actin cap dynamics (GFP:moeABD) and cell cycle (His:RFP) during the metaphase of cycle 10 to the interphase of cycle 11. Z-layer of actin and nucleus is 1 μm and 5 μm below the vitelline membrane, respectively. (F) Apical actin cap initiation (GFP:moeABD) and furrow dynamics (Gap43:mCh) at the end of cycle 11. (G) Apical actin displacement during cycle 10–13 (n = 4).
(TIF)

S2 Fig Nullo staining, aneuploid X and *nullo* Df furrow morphology, and ingression rate. (A) Aneuploid X and *nullo* Df furrow morphology in cycle 13, and z planes at -1 μm, -3 μm, -4 μm, -5 μm, -6 μm, -7 μm, and -8 μm. Scale bar = 5 μm. (B) Wild type, intact and deepest Aneuploid X and nullo Df furrow length for cycle 12 and 13 (WT: n = 7, Aneuploid X and nullo Df: n = 3). (C) Nullo protein localization in wild-type and *nullo* Df embryos in cycles 12–14. Anti-Nullo (green channel), F-actin (Palladian, red channel), and merged channel. Scale bar = 5 μm. (D) Wild type, intact and deepest Aneuploid X and nullo Df maximal furrow ingression rate in cycle 12 and 13 (WT: n = 7, Aneuploid X and nullo Df: n = 3).
(TIF)

S3 Fig Aneuploid 2L, 2R, 3L and 3R ingression I rates and cell cycle status. (A) Average furrow ingression I rates of WT, aneuploid 2L, and 2R embryos during cycles 11–13 (n≥3). *:p<0.05; *ns*: not significant. (B) Average furrow ingression I rates of WT, aneuploid 3L, and 3R embryos during cycles 11–13 (n≥3). *:p<0.05; *ns*: not significant. (C) Chromosomal morphologies indicate cell cycle status (Histone:GFP) at 0 min (interphase), 4 min (prophase), 8 min (metaphase), 10 min (anaphase), 12 mi (telophase), and 13 min (start of new cell cycle). Example nucleus from cycle 12, but similar morphologies are present during cycles 10–13. Arrowhead indicates the bright puncta in nuclei during prophase. Scale bar = 5 μm. (D) Furrow traces from individual *mei-41* mutant embryos demonstrate variable Stabilization timing and period, but possess reduced, separable Ingression I and Ingression II phases. (E) Furrow dynamics for zld RNAi (black curve) and zld RNAi + *mei41* mutation (blue curve) in Spider:GFP, His:RFP background (n = 4). (F) Maximal furrow length in zld RNAi and zld RNAi + *mei41* mutation during cycle 11–13 (n = 4). *: p<0.05; *ns*: not significant. (G) Maximal furrow ingression rate of zld RNAi and zld RNAi + mei41 mutation during cycle 11–13 (n = 4). *: p<0.05; *ns*: not significant. (H) Nuclear envelope staining in WT and *zld* mutant in cycle 13. Anti-Lamin (green channel), DAPI (blue channel), and merged channel are shown. Scale bar = 5 μm. (I) Adjacent nuclear fusion (upper panels) and mitotic nuclear fusion (bottom panels) phenotypes in α-amanitin injected embryo during cycle 13. Prophase, metaphase, anaphase, telophase and interphase in the next cycle are shown. Arrowheads indicate missegregated chromatins. Scale bar = 5 μm. (J) Intact furrow length and mitotic defects. The percentage of mitotic defects at cycle 13 in α-amanitin injected, *zld*, Aneuploid X, *nullo*, and WT embryos is plotted, as well as the intact furrow lengths during metaphase in cycle 13. (K) Interphase nuclear densities during cycles 10–13 (n = 4).
(TIF)
