# Supplementary figures and images for: Differentially-dimensioned furrow formation by zygotic gene expression and the MBT
Source: PLoS Genet. 2018 Jan 16;14(1):e1007174. doi: 10.1371/journal.pgen.1007174 (PMC5786337; doi:10.1371/journal.pgen.1007174)

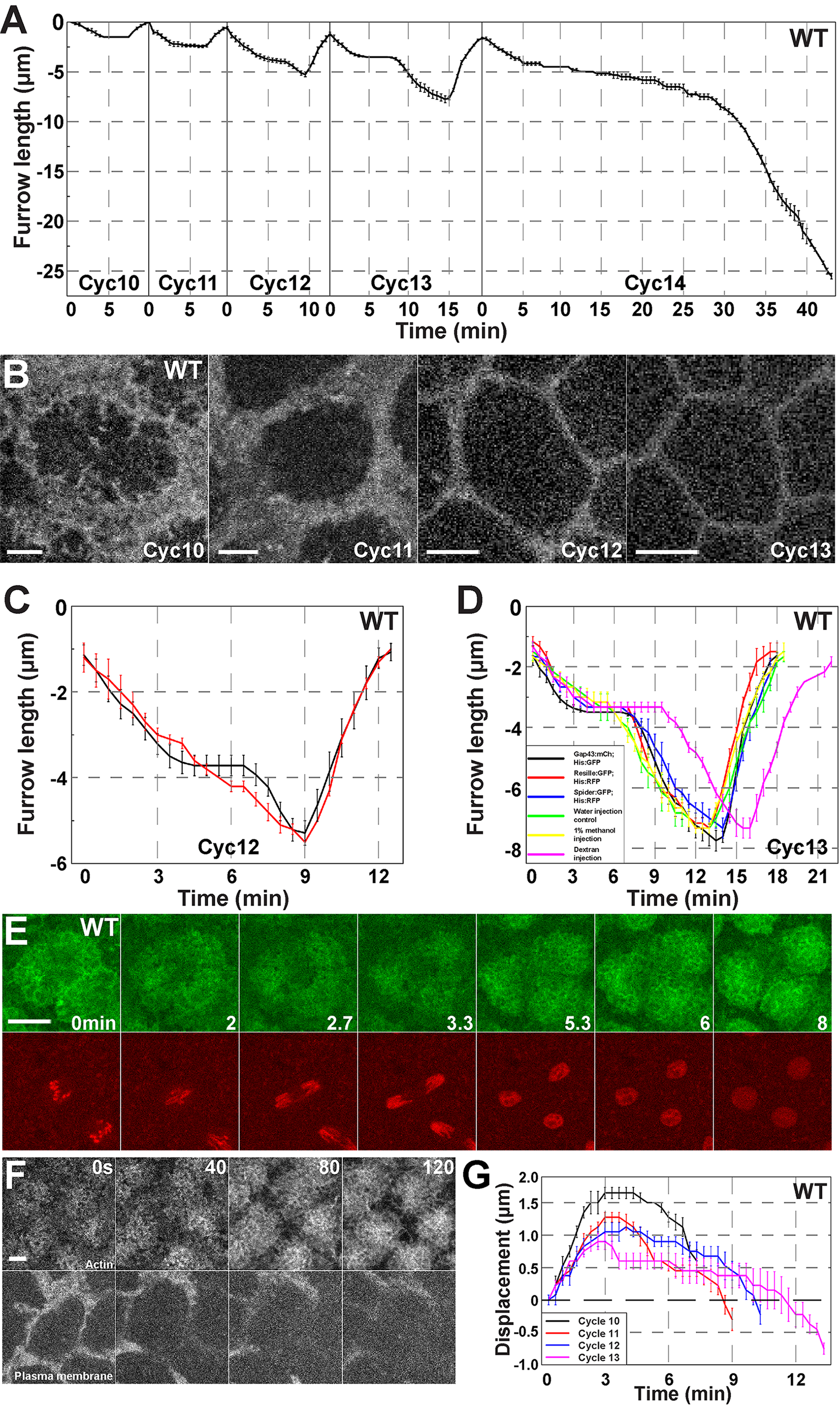

Supplement: S1 Fig — (A) Wild-type furrow dynamics, including cellularization, during cycles 10–14 (cycle 10: n = 4; cycle 11: n = 7; cycle 12 and 13: n = 8; cycle 14: n = 3). (B) Furrow morphology during cycles 10–13. Scale bar = 5 μm. (C) Wild type furrow dynamics from cycle 12. Red line shows the furrow dynamics without stabilization phase (n = 5 embryos); and black line shows with stabilization phase (n = 7 embryos). (D) Cycle 13 furrow dynamics for different membrane markers and injection controls (n≥3). (E) Apical actin cap dynamics (GFP:moeABD) and cell cycle (His:RFP) during the metaphase of cycle 10 to the interphase of cycle 11. Z-layer of actin and nucleus is 1 μm and 5 μm below the vitelline membrane, respectively. (F) Apical actin cap initiation (GFP:moeABD) and furrow dynamics (Gap43:mCh) at the end of cycle 11. (G) Apical actin displacement during cycle 10–13 (n = 4). (TIF) [file pgen.1007174.s001.tif]

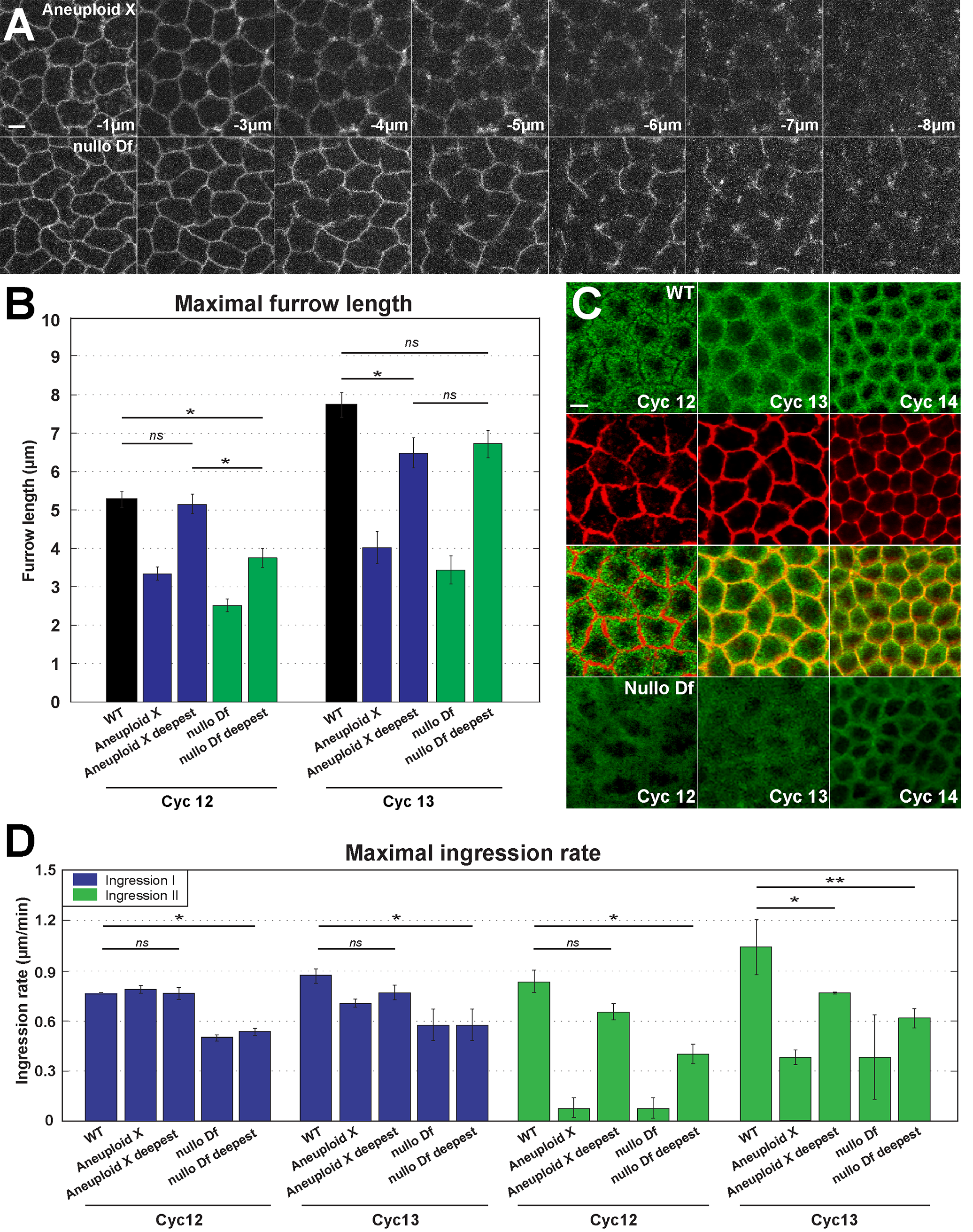

Supplement: S2 Fig — (A) Aneuploid X and nullo Df furrow morphology in cycle 13, and z planes at -1 μm, -3 μm, -4 μm, -5 μm, -6 μm, -7 μm, and -8 μm. Scale bar = 5 μm. (B) Wild type, intact and deepest Aneuploid X and nullo Df furrow length for cycle 12 and 13 (WT: n = 7, Aneuploid X and nullo Df: n = 3). (C) Nullo protein localization in wild-type and nullo Df embryos in cycles 12–14. Anti-Nullo (green channel), F-actin (Palladian, red channel), and merged channel. Scale bar = 5 μm. (D) Wild type, intact and deepest Aneuploid X and nullo Df maximal furrow ingression rate in cycle 12 and 13 (WT: n = 7, Aneuploid X and nullo Df: n = 3). (TIF) [file pgen.1007174.s002.tif]

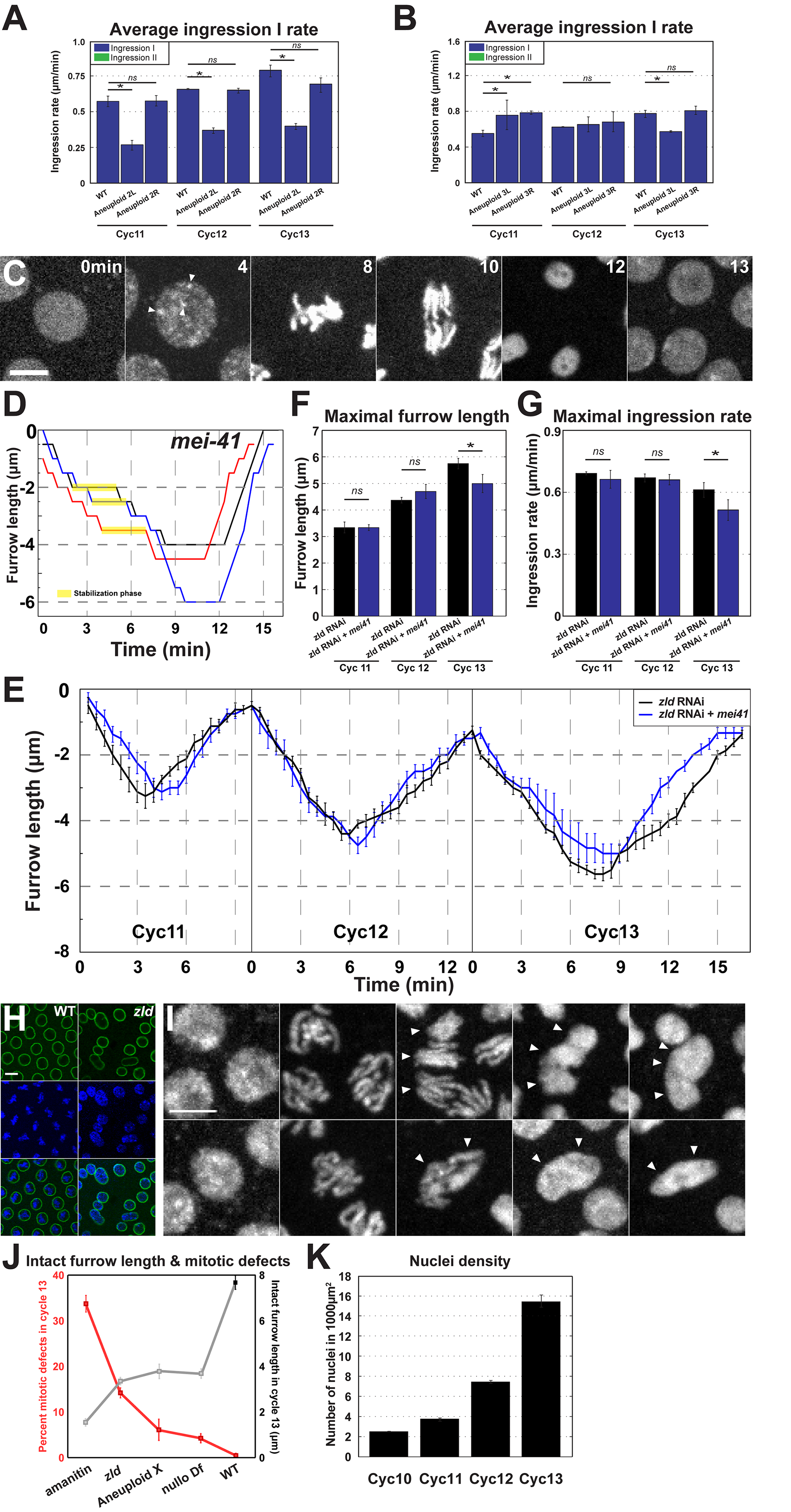

Supplement: S3 Fig — (A) Average furrow ingression I rates of WT, aneuploid 2L, and 2R embryos during cycles 11–13 (n≥3). *:p<0.05; ns: not significant. (B) Average furrow ingression I rates of WT, aneuploid 3L, and 3R embryos during cycles 11–13 (n≥3). *:p<0.05; ns: not significant. (C) Chromosomal morphologies indicate cell cycle status (Histone:GFP) at 0 min (interphase), 4 min (prophase), 8 min (metaphase), 10 min (anaphase), 12 mi (telophase), and 13 min (start of new cell cycle). Example nucleus from cycle 12, but similar morphologies are present during cycles 10–13. Arrowhead indicates the bright puncta in nuclei during prophase. Scale bar = 5 μm. (D) Furrow traces from individual mei-41 mutant embryos demonstrate variable Stabilization timing and period, but possess reduced, separable Ingression I and Ingression II phases. (E) Furrow dynamics for zld RNAi (black curve) and zld RNAi + mei41 mutation (blue curve) in Spider:GFP, His:RFP background (n = 4). (F) Maximal furrow length in zld RNAi and zld RNAi + mei41 mutation during cycle 11–13 (n = 4). *: p<0.05; ns: not significant. (G) Maximal furrow ingression rate of zld RNAi and zld RNAi + mei41 mutation during cycle 11–13 (n = 4). *: p<0.05; ns: not significant. (H) Nuclear envelope staining in WT and zld mutant in cycle 13. Anti-Lamin (green channel), DAPI (blue channel), and merged channel are shown. Scale bar = 5 μm. (I) Adjacent nuclear fusion (upper panels) and mitotic nuclear fusion (bottom panels) phenotypes in α-amanitin injected embryo during cycle 13. Prophase, metaphase, anaphase, telophase and interphase in the next cycle are shown. Arrowheads indicate missegregated chromatins. Scale bar = 5 μm. (J) Intact furrow length and mitotic defects. The percentage of mitotic defects at cycle 13 in α-amanitin injected, zld, Aneuploid X, nullo, and WT embryos is plotted, as well as the intact furrow lengths during metaphase in cycle 13. (K) Interphase nuclear densities during cycles 10–13 (n = 4). (TIF) [file pgen.1007174.s003.tif]
